# Evaluation of the Oral Health Education Programme for Nurses using an Oral Lesion Simulator

**DOI:** 10.3290/j.ohpd.b5458567

**Published:** 2024-06-12

**Authors:** Satoru Haresaku, Toru Naito, Maki Miyoshi, Hisae Aoki, Mayumi Monji, Ayako Nishida, Yoshinori Kono, Maiko Kayama, Yojiro Umezaki

**Affiliations:** a Professor, Department of Nursing, Fukuoka Nursing College, Fukuoka, Japan. Searched and reviewed the literature, analysed the data, and wrote the manuscript.; b Professor, Section of Geriatric Dentistry, Department of General Dentistry, Fukuoka Dental College, Fukuoka, Japan. Designed and created simulators with oral disease and symptoms, contributed to creation of the educational programmes.; c Associate Professor, Department of Nursing, Fukuoka Nursing College, Fukuoka, Japan. Participated in the educational programmes, instructed the nursing students, assisted in finding documents and issuing the questionnaires, contributed to analysing the data, examined the manuscript.; d Professor, Department of Nursing, Fukuoka Nursing College, Fukuoka, Japan. Critically reviewed the manuscript, supervised the entire study process.; e Associate Professor, Department of Nursing, Fukuoka Nursing College, Fukuoka, Japan. Participated in the educational programmes, instructed the nursing students, assisted in finding documents and issuing the questionnaires, contributed to analysing the data, examined the manuscript.; f Assistant Professor, Department of Nursing, Fukuoka Nursing College, Fukuoka, Japan. Participated in the educational programmes, instructed the nursing students, assisted in finding documents and issuing the questionnaires, contributed to analysing the data, examined the manuscript.; g Associate Professor, Section of Geriatric Dentistry, Department of General Dentistry, Fukuoka Dental College, Fukuoka, Japan. Designed and created simulators with oral disease and symptoms, contributed to creation of the educational programmes.; All authors read and approved the manuscript.

**Keywords:** collaborative education, nursing student, oral assessment, Oral Health Assessment Tool, oral simulator

## Abstract

**Purpose:**

This study aimed to investigate the usefulness of a newly developed oral simulator for nursing students’ oral assessment education on oral diseases and symptoms.

**Materials and Methods:**

The participants were first-year students (n=105) at a nursing school in Japan. Ten identical oral simulators with angular cheilitis, missing teeth, dental caries, calculus, periodontitis, hypoglossal induration, food debris, and crust formation were created by a team of dentists. After a 45-minute lecture programme for oral assessment performance with the Oral Health Assessment Tool (OHAT), the ability test with the simulators and the OHAT as well as test feedback were conducted in a 30-minute practical programme. To evaluate the effectiveness of the programmes, questionnaires and ability tests with slides of oral images were conducted at baseline and after the programme.

**Results:**

Ninety-nine students (94.3%) participated in this study. The results of the ability test with the simulators and the OHAT in the practical programme showed that the correct answer rates of assessing tongue, gingiva, present teeth, and oral pain were less than 40%. Their levels of confidence, perception, and oral assessment performance were statistically significantly higher after the programmes than they were at baseline. Their level of confidence in assessing the need for dental referral had the largest increase in scores compared to the lowest scores at baseline in the nine post-programme assessment categories.

**Conclusions:**

This study identified several problems with nursing students’ oral assessment skills and improvements of their oral assessment confidence, perceptions and performance.

Elderly people with mental illnesses such as dementia are more likely to have severe oral diseases than healthy elderly people.^7,14^ The number of people with severe oral diseases will increase in the future, as the number of people with dementia worldwide is expected to increase from 55 million in 2020 to 139 million in 2050.^29^ However, there is limited access to oral health care for this population due to lack of motivation on the part of dental professionals, as well as apathy, limited cooperation, low adaptability to new prostheses, fear of treatment, poor communication and mobility difficulties among those patients.^4,15,30^

A previous systematic review regarding advanced dental disease in people with severe mental illness suggested that their management should include oral assessment using standard checklists that can be completed by non-oral healthcare professionals.^14^ Oral assessment tools have been developed and used to enable non-oral healthcare professionals to easily perform oral assessments.^2,6^

Nurses play an important role in caring for such patients and performing oral assessments, dental referrals through physicians, and collaborative oral healthcare practices with oral healthcare professionals.^11,18,26^ A previous study reported that their higher oral assessment performances were more likely to encourage their patients to see a dentist.^11^ Therefore, nursing students should be educated and qualified on oral assessment to improve their performance concerning patients and dental referral.

However, some studies reported that nurses had limited oral healthcare knowledge and inadequate understanding of the crucial elements of an oral health assessment.^3,11^ In addition, a recent study reported that nursing students could not perform student-on-student and patient-based training in oral assessment and healthcare owing to the risk of SARS-CoV-2 transmission.^10^ This problem may have a negative impact on nursing students’ oral assessment performance and collaboration with oral healthcare professionals after their qualification.

To address these problems, an oral simulator with oral diseases and symptoms was developed for nursing oral-assessment education. Only a few studies exist on the use of oral simulators for nursing oral assessment education, although some studies have reported that nursing students’ oral assessment performance was improved by interprofessional education and collaborative education between nurses and oral healthcare professionals.^5,9,12^

This study aimed to investigate the usefulness of the new oral simulator with oral diseases and symptoms for nursing students’ oral assessment education to promote their collaboration with oral healthcare professionals after qualification.

## Materials and Methods

### Ethical Approval

All the procedures involving human participants in this study were approved by the Ethics Committee of Fukuoka Gakuen, Fukuoka, Japan (approval No. 596) and were in accordance with the Ethical Guidelines for Clinical Research (the Ministry of Health, Labour and Welfare, Tokyo, Japan, No. 415 of 2008) and the 1964 Declaration of Helsinki and its later amendments or comparable ethical standards.

### Design and Sample

**Fig 1:**
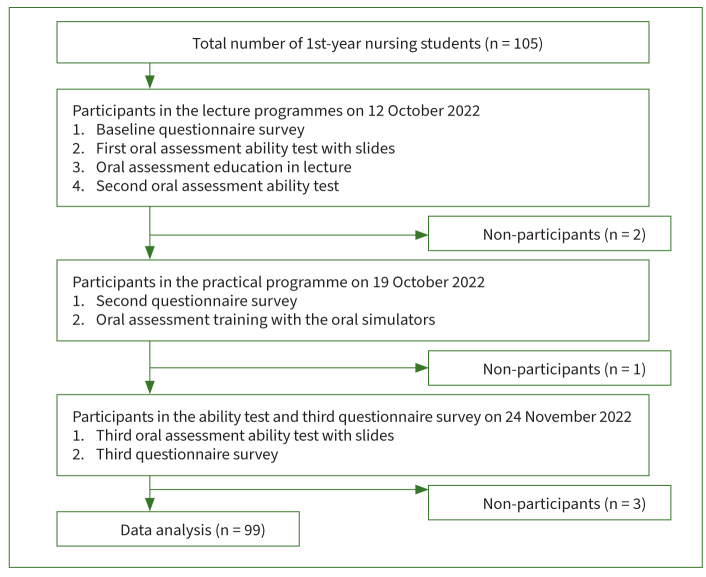
Flow chart illustrating the selection of study participants.

This study was a before-and-after survey with oral simulators that simulated oral disease and symptoms. The participants were first-year nursing students (n=105) from a nursing school in Fukuoka city, Japan. An oral assessment programme with oral simulators and the Japanese version of the ORAL HEALTH ASSESSMENT TOOL (OHAT)^17^ was developed in 2022. The lecture and practical programmes were conducted on 12 and 19 October 2022, respectively (Fig 1). To evaluate the effectiveness of the programmes, questionnaires and ability tests were conducted at baseline, after the lecture programme, and after the practical programme (Fig 1).

### Oral Simulator with Oral Diseases and Symptoms

**Fig 2:**
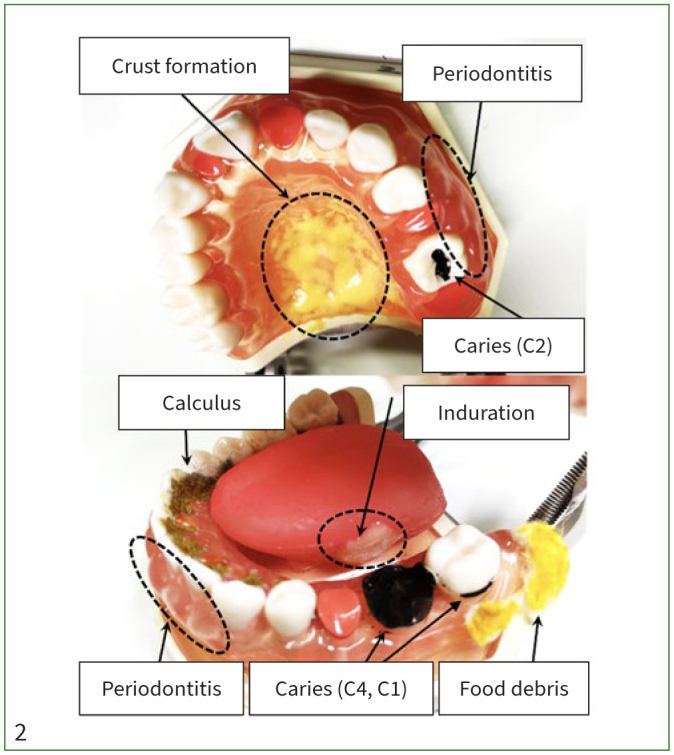
Oral simulator with dental disease and symptoms.

**Fig 3:**
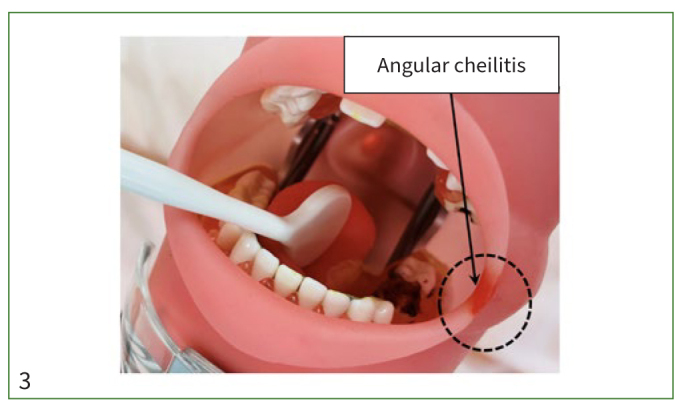
Oral simulator set in a mannequin.

Ten identical oral simulators with missing teeth, dental caries, calculus, periodontitis, induration of the sublingual region, food debris, and crust formation on the palate (Fig 2) were made by 2 dentists who were affiliated with the department of geriatric dentistry in a dental school. The simulators had 3 carious lesions (26, 36, 37) and 5 missing teeth (12, 21, 25, 27, 35). Induration of the sublingual region and crust formation on the palate were made from resin, and food debris was made from cotton with yellow ink. Artificial dental calculus (Nissin; Tokyo, Japan) was placed on the lingual aspect of mandibular teeth. The simulators were wrapped in artificial oral mucosa (Nissin) with red ink on the left side of the angle of the mouth representing angle cheilitis and set in the manikins, which were placed on the hospital bedside table (Fig 3).

### Lecture and Practical Programmes

In the 45-minute lecture programme, a dentist specialising in preventive dentistry instructed the nursing students on oral assessment with the OHAT sheet. The sheet has the images of the Face Scale in the oral pain category and oral status photographs in the other assessment categories according to scores so that non-oral professionals can easily choose the scores.^17^ In the programme, the students were instructed to assess oral status using the images on the sheet; to choose the status as healthy (0), signs of alteration (1), or unhealthy (2); assess the need for dental referral as no need (0) or need (1); and complete those scores on the sheet. In addition, students were instructed to choose a score of 2 (unhealthy) in the saliva category if they found crust formation on the palate.

In the practical programme, the 30-minute oral assessment and 30-minute oral healthcare performance programmes were conducted under the instruction and supervision of the dentists and 3 nurses. First, an oral assessment ability test with the simulators was conducted in four student groups. The students in the group, one by one for 5 minutes each, assessed the oral simulator with a dental mirror and the OHAT sheets. After all groups finished their assessments, the instructors provided feedback. After completing the oral assessment programme, the oral healthcare programme was conducted.

### Ability Tests with the Oral Simulator and Slides of Oral Images

Tests using the oral simulator and slides of oral images were conducted to evaluate the students’ ability to assess oral status. The OHAT sheets were distributed to the students before the tests.

The test with the oral simulator was conducted in the practical programme. First, the students counted the number of teeth present (excluding wisdom teeth), missing teeth, and decayed teeth in the oral simulator and wrote the numbers on a form. Then they assessed the oral status of the simulators and entered the scores on the OHAT sheet. The numbers and scores for correct answers by dentists were compared to the students’ answers, and the percentages of correct answers according to the numbers and scores in the tests were calculated.

The slide tests were conducted at baseline, after the lecture programme, and after the practical programme. The eight oral status images to assess each of the eight assessment categories were projected sequentially for 30 s on an LCD screen. During the projection of the images, the students assessed the images, scored them, and noted the scores on the sheets. After the projection, they assessed the need for dental referral and noted the score on the sheet. The percentages of correct answers according to the oral assessment categories in the tests and the total number of correct answers (0–9) were calculated.

### Structured Questionnaires

The questionnaire was based on a previously developed questionnaire used in an earlier study to investigate the effectiveness of oral assessment education programmes.^9^ The questionnaire consisted of 3 parts: characteristics (sex and age), confidence in oral assessment, and perceptions regarding oral assessment.

Regarding confidence in oral assessment, the students were asked about their confidence in assessing oral status in each oral assessment category. A 5-point Likert scale was used, with confidence scores ranging from 1 (no confidence) to 5 (strong confidence). A mean score of 3 points or more was considered to indicate confidence, and a score <3 points was considered to indicate no confidence.

Regarding perceptions of oral assessment, the students were asked about their level of agreement with the statements that (1) nurses can assess the oral status of their patients, (2) nurses should perform oral assessments, and (3) they hope to perform oral assessments. A 5-point Likert scale was used that included strongly agree, agree, neither agree nor disagree, disagree, or strongly disagree; the attitude scores ranged from 1 (strongly disagree) to 5 (strongly agree).

Cronbach’s alpha was used to assess the reliability of the questionnaire. Cronbach’s alpha of the questionnaire in the domains of confidence and perceptions of oral performance were 0.811 and 0.933, respectively.

### Data Procedure

The questionnaire data were collected on 12 October, 19 October, and 24 November 2022; the oral simulator test data were collected on 19 October. The data from the slide test were collected on 12 October before and after the lecture programme and on 24 November after the practical programme (Fig 1). The consent form was distributed to the students before the lecture programme. They were informed about the study by the researchers and asked to sign the consent form if they agreed to participate.

### Statistical Analysis

A chi-squared test was used to compare the correct answer rates in the ability test with the slides between baseline and after the lecture programme, between post-lecture and the practical programme, and between baseline and the practical programme.

A Wilcoxon signed-rank test was used to compare the levels of confidence and perceptions of oral assessment between baseline and the post-lecture programme and between post-lecture and the practical programme.

The level of statistical significance was set at 5%. The statistical analyses were performed using the IBM SPSS Statistics software program (Version 21.0; IBM; Armonk, NY, USA).

## Results

Ninety-nine students (87 females and 12 males) participated in this study. The participation rate was 94.3%.

Table 1 shows the range and distribution of the number and score selected by the nursing students in the oral assessment ability tests with oral simulators in the practical programme. The numbers of present, missing, and decayed teeth selected by dentists (number of correct answers) were 23, 5, and 3, respectively, and the ranges of the numbers selected by the nursing students were 20-24, 1-8, and 0-7, respectively. The percentages of correct answers in present, missing, and decayed teeth were 83.8%, 66.7%, and 59.6%, respectively. In the assessment categories, more than 60% of the students assessed lip, saliva, oral cleanliness, and need for dental referral correctly; however, less than 40% assessed tongue and tongue coating, gingiva and oral mucosa, present teeth, and oral pain correctly. Approximately half of them selected a score of 0 (healthy) in the category of tongue and tongue coating, and 16.2% did so in the category of gingiva and oral mucosa, although the correct scores were 2 (unhealthy).

**Table 1:** Range and distributions of numbers and scores in the oral assessment ability tests with oral simulators

Number of teeth	CA*	Range**	Distribution of the selected number (%)		
			<CA*	CA*	>CA*
Number of present teeth	23	20-24	11.1%	83.8%†	5.1%
Number of missing teeth	5	1-8	17.2%	66.7%†	16.2%
Number of decayed teeth	3	0-7	38.4%	59.6%†	2.0%
Assessment category	CA*	Range**	Distribution of the selected score (%)		
			Score 0	Score 1	Score 2
Lip	1	0-2	7.1%	78.8%†	14.1%
Tongue and tongue coating	2	0-2	49.5%	26.3%	24.2%†
Gingiva and oral mucosa	2	0-2	16.2%	47.5%	36.4%†
Saliva	2	0-2	14.1%	25.3%	60.6%†
Present teeth	1	1, 2	0.0%	36.4%†	63.6%
Removable dentures	0	0-2	88.9%†	6.1%	5.1%
Oral cleanliness	2	1, 2	0.0%	10.1%	89.9%†
Oral pain	0	0-2	28.3%†	19.2%	52.5%
Need for dental referral	1	1	0.0%	100.0%†	-

*Correct answers determined by dentists; **range of numbers or scores selected by the nursing students; †percentage of correct answers. <CA = fewer correct answers than by dentists; >CA = more correct answers than by dentists.

Table 2 shows the percentages and the mean number of correct answers in the oral assessment ability tests with slides between the baseline and post-programme timepoints. Less than 60% of them correctly assessed lip, tongue and tongue coating, gingiva and oral mucosa, saliva, and removable dentures at baseline. The percentages of correct answers increased statistically significantly in five assessment categories after the practical programme compared to baseline (p < 0.05). The mean number of correct answers also increased statistically significantly after both the lecture and practical programmes (p < 0.05).

**Table 2:** Comparisons of percentages and mean number of correct answers in the ability tests with slides between the baseline and post-programme timepoints

Assessment category	CA*	Baseline (A)	After lecture (B)	After practical programme (C)	A vs B	B vs C	A vs C
		(%)	(%)	(%)	p-value**	p-value**	p-value**
Lip	2	52.5%	64.6%	70.7%	0.083	0.362	0.009
Tongue and tongue coating	1	59.6%	68.7%	54.5%	0.182	0.041	0.473
Gingiva and oral mucosa	2	46.5%	76.8%	80.8%	<0.001	0.487	<0.001
Saliva	2	41.4%	74.7%	83.8%	<0.001	0.114	<0.001
Present teeth	2	94.9%	97.0%	98.0%	0.470	0.651	0.248
Removable dentures	2	53.5%	63.6%	78.8%	0.149	0.019	0.000
Oral cleanliness	2	91.9%	97.0%	94.9%	0.121	0.470	0.389
Oral pain	2	61.6%	67.7%	75.8%	0.372	0.207	0.032
Need for dental referral	1	99.0%	100.0%	100.0%	0.316	-	0.316
		Baseline (A)	After lecture (B)	After practical programme (C)	A vs B	B vs C	A vs C
		Mean±SD	Mean±SD	Mean±SD	p-value†	p-value†	p-value†
Mean number of correct answers		6.0±1.5	7.1±1.2	7.4±1.2	<0.001	0.040	<0.001

*Correct answer determined by dentists; **chi-squared test; †Wilcoxon signed-rank test.

Table 3 shows a comparison of the nursing students’ confidence level in performing oral assessments according to assessment category between the baseline and post-programme timepoints. The mean confidence levels were >3 in all assessment categories after the practical programme, although they were <3 at baseline. The level of assessing the need for dental referral was the highest in the assessment categories after the practical programme, although it was the lowest at baseline. The confidence levels were statistically significantly higher in all assessment categories after both the lecture and practical programmes (p < 0.05).

**Table 3:** Comparisons of confidence level* of performing oral assessment between the baseline and post-programme timepoints

Assessment category	Baseline (A)	After lecture (B)	After practical programme (C)	A vs B	B vs C	B vs C
	Mean±SD	Mean±SD	Mean±SD	p-value**	p-value**	p-value**
Lip	2.8±1.1	3.3±1.0	3.7±0.9	<0.001	0.001	<0.001
Tongue and tongue coating	2.5±1.0	3.2±0.9	3.4±0.9	<0.001	0.021	<0.001
Gingiva and oral mucosa	2.5±1.1	3.0±1.0	3.3±1.0	<0.001	0.001	<0.001
Saliva	2.3±1.1	2.9±1.1	3.4±1.0	<0.001	<0.001	<0.001
Present teeth	2.5±1.0	3.1±1.0	3.6±0.9	<0.001	<0.001	<0.001
Removable dentures	2.8±1.3	3.3±1.3	3.8±1.0	<0.001	<0.001	<0.001
Oral cleanliness	2.7±0.9	3.0±0.9	3.6±0.9	0.002	<0.001	<0.001
Oral pain	2.7±1.2	3.2±1.1	3.6±0.9	<0.001	<0.001	<0.001
Need for dental referral	2.3±1.0	3.0±1.1	3.9±1.0	<0.001	<0.001	<0.001

*No confidence=1; strong confidence=5; **Wilcoxon signed-rank test.

Table 4 shows a comparison of nursing students’ perception level regarding oral assessment performance between the baseline and post-programme timepoints. The mean perception levels were >4 in all categories after the practical programme, although they were <4 in four categories at baseline. The levels of nurses’ perception that they can assess the status of oral hygiene, dental caries, periodontal disease, and oral cancer were statistically significantly higher after the practical programme than after the lecture programme (p < 0.05). The levels of nurses’ perceptions that they should encourage their patients with dental problems to see a dentist and that they hope to perform oral assessment and encourage patients with dental problems to see a dentist after qualification were statistically significantly higher after the programmes compared to baseline (p < 0.05).

**Table 4:** Comparisons of perception levels* regarding oral assessment performance between the baseline and post-programme timepoints

Category	A: baseline	B: after lecture	C: after practical programme	A vs B	B vs C	A vs C
	Mean±SD	Mean±SD	Mean±SD	p-value**	p-value**	p-value**
Nurses can assess the status of oral hygiene in their patients	4.0±0.7	4.0±0.8	4.2±0.7	0.749	0.015	0.058
Nurses can assess the presence of dental caries in their patients	3.5±1.0	4.0±0.9	4.3±0.7	0.000	0.000	0.000
Nurses can assess the presence of periodontal disease in their patients	3.5±1.0	3.7±0.8	4.0±0.9	0.120	0.005	0.001
Nurses can assess the presence of oral cancer in their patients	3.4±1.2	3.6±1.0	4.0±1.0	0.177	0.000	0.000
Nurses should perform oral assessment to provide appropriate oral healthcare for their patients	4.3±0.9	4.3±0.9	4.5±0.7	0.610	0.112	0.350
Nurses should encourage their patients with dental problems to see a dentist	4.2±0.9	4.4±0.8	4.5±0.7	0.017	0.091	0.000
I hope that I will perform oral assessment for my patients after qualification	3.9±0.9	4.1±0.9	4.1±0.9	0.012	0.873	0.008
I hope that I will encourage patients with dental problems to see a dentist	4.1±0.9	4.3±0.8	4.3±0.8	0.001	0.635	0.029

*Strongly disagree=1; strongly agree=5; **Wilcoxon signed-rank test.

## Discussion

This study is the first to develop oral simulators with oral disease and symptoms for nursing oral-assessment education and to investigate the effectiveness of their use in nursing education.

The present study revealed several problems regarding nursing students’ abilities in oral assessment performance in the test with the simulators. Approximately one-third of them could not count the correct number of missing teeth, and 40% could not count that of decayed teeth in the practical programme after the lecture programme. Caries is the main cause of tooth loss^24^ and contributes to oral hypofunction,^21^ frailty,^27^ dementia,^20^ and pneumonia mortality^23^ among elderly people. Therefore, they should be educated about counting them correctly so that they can refer patients with caries and missing teeth to dentists to provide dental treatment to the patients.

The oral simulators had an 8-mm-diameter induration model in the sublingual region, and a score of 2 was selected in the assessment category of tongue and tongue coating. However, approximately half of them selected a score of 0, and the results indicated that they did not recognise induration. Oral mucosal cancer may appear as an indurated raised nodule, often with an ulcerated surface that may cause little pain.^28^ The most common subsite of oral squamous cell carcinoma is the oral tongue, and the estimated frequency of oral tongue squamous cell carcinoma is 60%.^1^ Therefore, nursing students should trained in identifying and assessing induration so that they can immediately refer the patient to a dentist (after their qualification). In addition, the ability to assess the tongue and tongue coating did not improve statistically significantly in the ability tests with the slides of oral images. Mild tongue coating on the tongue dorsum was in the oral image and the students did not seem able to assess it correctly; therefore, the programmes should be improved so that students can assess tongue coating correctly.

The oral simulators had gingivitis in the whole maxillary and mandibular periodontal tissue. However, approximately two-thirds of the students could not correctly assess the components of the category of gingiva and oral mucosa, and approximately 16% of the students assessed the gingiva and oral mucosa as healthy. The results suggested that it might be difficult for them to assess the periodontal condition. Periodontal disease affects systemic illnesses, such as cardiovascular disease^16^ and diabetes.^19^ Therefore, programmes for the assessment of periodontal conditions should be enhanced using oral simulators, images, and real patients.

The simulators had a crust formation model on the palate. The students were taught in the lecture programme that it is caused by xerostomia and that the score in the assessment category of saliva is 2.^13^ As a result, approximately 60% of them could assess the category of saliva correctly. However, as it is impossible to measure the quantity of saliva in simulators, training programmes with images, videos, and real patients should be developed to enable them to assess the salivary status in clinical settings.

Approximately half of the students assessed mannequins with normal facial expressions as unhealthy. The Wong-Baker Faces Pain Rating Scale is widely used to assess pain in children and elderly individuals because it is easy to understand, using a series of face images ranging from a happy face at 0, or “no hurt”, to a crying face at 10.^22^ Therefore, programmes including the use of this scale might be effective in improving the ability to assess oral pain.

Students’ abilities, confidence, and perceptions of assessing oral status and the need for dental referral improved statistically significantly through those programmes. A previous study showed that nurses’ positive oral assessment performances were associated with their positive encouragement of patients to see dentists.^11^ Therefore, the improvements might contribute to promoting their oral assessment performance and dental referral for their patients in clinical practice.

In Japan, the use of oral assessment tools by nurses is not widespread,^11^ and less than one-fourth of nursing schools in Japan conduct oral assessment education with the tools.^8^ Therefore, it is suggested that oral healthcare professionals should support nursing assessment education to promote students’ ability to correctly assess dental diseases and symptoms, make dental referrals, and provide collaborative oral healthcare with oral healthcare professionals. Dentists can make well-designed oral simulators with oral disease and symptoms to support this training.

There are several limitations associated with this study. First, as the participants were all first-year students, further studies are needed to continuously investigate whether the improvement of abilities, confidence, and perceptions regarding oral assessment performance can contribute to nursing students’ performances in clinical practice.

A control group was not included in this study because all students needed to take these programmes at the same time. A previous study reported that oral assessment education with both a lecture programme and a student-on-student programme was effective in improving abilities, confidence, and perceptions.^9^ However, most students in the present study did not have severe dental diseases or symptoms. In addition, a recent study regarding nursing education reported that student-on-student training could not be conducted at nursing schools, including the investigated school, owing to the risk of SARS-CoV-2 transmission.^10^ Therefore, it is suggested that oral assessment programmes with oral simulators may be more effective and useful for assessing oral diseases than student-on-student training during the spread of COVID-19 or pandemic conditions.

The values of domains in the questionnaire of the present study were reliable, as a Cronbach’s alpha of >0.7 was considered reliable.^25^

A limitation of the study was that the effectiveness of oral assessment education programmes with oral simulators was investigated in only one nursing school in Japan. Further studies in other nursing schools should be conducted.

## Conclusions

This study identified several problems with nursing students’ oral assessment skills and improvements of their oral assessment confidence, perceptions, and performance. Further studies are needed to investigate such programmes in other populations to assess their effectiveness in other nursing schools worldwide.
